# Supplementary figures and images for: The experience of the COVID-19 pandemic by persons with ASD: Social aspects
Source: PLoS One. 2022 Jun 16;17(6):e0267123. doi: 10.1371/journal.pone.0267123 (PMC9202849; doi:10.1371/journal.pone.0267123)

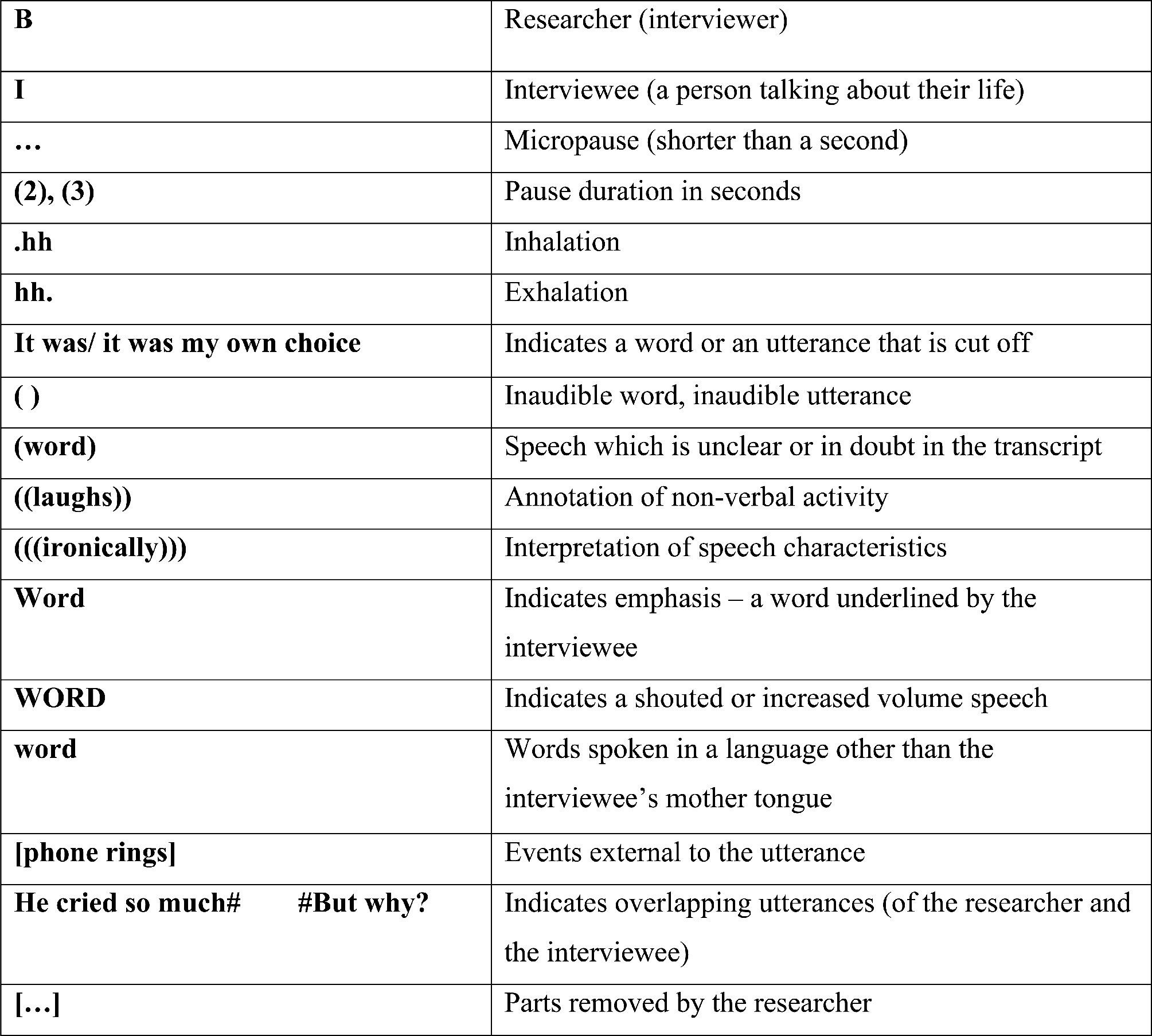

Supplement: S1 Table — (TIF) [file pone.0267123.s001.tif]
