# Supplementary material for: The experience of the COVID-19 pandemic by persons with ASD: Social aspects
Source: PLoS One. 2022 Jun 16;17(6):e0267123. doi: 10.1371/journal.pone.0267123 (PMC9202849; doi:10.1371/journal.pone.0267123)
Supplement: Attachment — Submitted filename: Reviewers Comments-Mohandas.docx [file pone.0267123.s002.docx]

Reviewer’s Comments

The Experience of the COVID-19 Pandemic by Autistic People: Social Aspects

--Manuscript Draft--

Manuscript Number: PONE-D-21-24954

Covid 19 pandemic has resulted in multiple psychological problems-(boredom, frustration, and embitterment), psychiatric issues (anxiety, depression, post-traumatic stress disorder, suicidal behavior and acute psychotic episodes) and social consequences (stigma, financial instability, sense of isolation etc).Studies of Covid 19 pandemic related issues have been addressed on people with Autistic Spectrum Disorder and their care givers(Yasser SaeedKhan et al,2021; Raman Baweja et al 2021, JorgeLugo-Marín et al (2021)).Several strategies have been advanced including Pfeiffer’s team in the college’s REACH Lab (Research, Engagement and Advocacy for Community Participation and Health) interventions and outcome measures aimed at promoting inclusion in community activities for those with ASD and intellectual and developmental disabilities (IDD).

This qualitative research has employed double hermeneutic of Anthony Giddens and interpretative phenomenological analysis (combination of psychological, interpretative, and idiographic components) with eight issues in ten adults diagnosed with autism spectrum, aged 25–45, out of which two as non-binary or genderqueer .Jefferson Transcription System was used in assessing participants’ statements (five recurring topics).The thematic analysis usually involves coding reliability thematic analysis, codebook thematic analysis and reflexive thematic analysis.

The case analysis reveals ‘the sense of falling out of social roles’, suicidal preoccupation, ‘craved for some interaction, but on the other hand, direct contact with people during the pandemic made her anxious’.

Digital health measures are necessary in this changing scenario as exemplified by Baweja’s article. The current article suggests ‘many autistic people seem to be well prepared for functioning in the new, rapidly emerging digital reality’. This may be true especially in high functioning autistic individuals

Additional Comments

1 Qualitative research needs small sample as in this article

2 The authors have employed probably the best methodological approach during the difficult times of covid

3 These digital methods may be incorporated into the frame work of ASD, especially in high

functioning cases

4 Further qualitative researches may help in adopting new strategies

## (Yasser SaeedKhan et al (2021) [Research in Developmental Disabilities](https://www.sciencedirect.com/science/journal/08914222) 119:104090;

## Raman Baweja et al (2021) *Journal of Autism and Developmental Disorders*;

## JorgeLugo-Marín et al (2021) [Research in Autism Spectrum Disorders](https://www.sciencedirect.com/science/journal/17509467)83, 101757)

[Reviewer: Dr Mohandas Warrier, Sun Medical and Research Centre, Trichur, Kerala 680002
