# Supplementary material for: The experience of the COVID-19 pandemic by persons with ASD: Social aspects
Source: PLoS One. 2022 Jun 16;17(6):e0267123. doi: 10.1371/journal.pone.0267123 (PMC9202849; doi:10.1371/journal.pone.0267123)
Supplement: Attachment — Submitted filename: Responses to Reviewers.docx [file pone.0267123.s003.docx]

# **Response to Reviewers**

## # Reviewer 1

Dear Prof. Das,

We would like to express our sincere gratitude for instructive comments. We have incorporated most of the suggestions. Those changes are highlighted within the manuscript. Please see below, for a point-by-point response to comments. All page numbers refer to the revised manuscript file with tracked changes.

### Reviewer comments to the Authors:

**a.** It would be better to avoid terms like “autistic people”. It sounds more stigmatizing. It would be better, if the authors use the term “persons with autism spectrum disorders”.

**Authors’ response:** In the manuscript, we deliberately used Identity-First Language, which is recommended by ASAN:

<https://autisticadvocacy.org/about-asan/identity-first-language/>

<https://autisticuk.org/wp-content/uploads/2016/05/AUTISTIC-UK-KEY-TEXTS-1-WHY-I-DISLIKE-PERSON-FIRST-LANGUAGE.pdf>

<https://www.ncbi.nlm.nih.gov/pmc/articles/PMC7817071/>

Our team consists of autistic self-advocates researchers (*Jasna Strona Spektrum,* ASAN). If it is necessary to change the language, we agree, but Identity-First Language is not stigmatizing for us and we use it in our work and private life.

**[on page 1, Title]**

The current change title is in line with medical nomenclature.

**b.** Two different abstracts (one structured and one unstructured) are used by the authors. Kindly check.

**Authors’ response:** Thank you for pointing this out. The reviewer is correct. Sending the abstract and lay abstract results from our mistake.

**c.** What is the hypothesis of the study?

**Authors’ response:** In qualitative social research, we do not begin the investigation from hypotheses („null hypothesis”). Research questions are formulated on the basis of the aims.

**d.** It is well known that significant proportion of people with autism spectrum disorders, have intellectual disability. Whether the authors considered the IQ of the participants before conducting interview and obtaining consent? Presence of psychopathology and severity of autism may influence the engagement in interview for assessment. This may influence the results significantly. How, these factors are taken care?

**Authors’ response:** Thank you, this is an important point. Many people on the autism spectrum suffer from anxiety disorders and depression, but we took this fact into consideration <https://link.springer.com/article/10.1007/s40489-021-00267-6>

We recruited participants through organizations offering diagnosis and therapy for autistic people and in order for the qualification to the sample group we conducted a self-report poll were criterion was the intellectual norm. The participants' consent was voluntary and self-conscious.

**[on page 3, lines 91 to 95]**

“All participants are within the intellectual norm or have supra-normative cognitive abilities. Like many people on the autism spectrum, they may have anxiety disorders or depression [13, 14, 15].

They were qualified on the basis of self-report, but their intellectual status was confirmed by the organizations that made contacts with each of them available.”

## # Reviewer 2

Dear Dr Warrier,

Thank you for your positive review. We would like to express our sincere gratitude for instructive comments. Those changes are highlighted within the manuscript. All page numbers refer to the revised manuscript file with tracked changes.

### Reviewer comments to the Authors:

**a, b.** May include the recent articles, may specify high functioning ASD if necessary. (…)

**Authors’ response:** Thank you for this suggestion. In line with the recommendation, we extended the references to include the recent articles:

**[on page 3, lines 91 to 93]**

“All participants are within the intellectual norm or have supra-normative cognitive abilities. Like many people on the autism spectrum, they may have anxiety disorders or depression [13, 14, 15].”

**[on page 19, lines 543 to 553]**

13. Baweja R., Brown S., Edwards E., Murray M. (2021), COVID-19 Pandemic and Impact on Patients with Autism Spectrum Disorder. Journal of Autism and Developmental Disorders, doi: 10.1007/s10803-021-04950-9

14. Lugo-Marín J., Gisbert-Gustemps L., Setien-Ramos I., Español-Martín G., Ibañez-Jimenez P., Forner-Puntonet M. et al (2021), COVID-19 pandemic effects in people with Autism Spectrum Disorder and their caregivers: Evaluation of social distancing and lockdown impact on mental health and general status. Research in Autism Spectrum Disorders 83:101757, doi: 10.1016/j.rasd.2021.101757

15. Khan Y.S., Khan A.W. Tahir M.E., Hammoudeh S., Shamlawi M.A., Alabdulla M. (2021), The impact of COVID-19 pandemic social restrictions on individuals with autism spectrum disorder and their caregivers in the State of Qatar: A cross-sectional study. Research in Developmental Disabilities 119:104090, doi: 10.1016/j.ridd.2021.104090

**Best regards**,

Authors
